# Book Reviews

**Published:** 1800-10

**Authors:** 


### CRITICAL RETROSPECT
### OF
### MEDICAL AND PHYSICAL LITERATURE.
### [ FOREIGN AND DOMESTIC. ]

*A Treatife on the Chemical Hiftory and Medical Powers of fome of the moft celebrated Mineral Waters; with Practical Remarks on the Aqueous Regimen; to which are added, Obfervations on the Ufe of Cold and Warm Bathing.* By William Saunders, M.D. F.R.S. 8vo. pp. 483, price 8s. in boards. London, 1800, W. Phillips.

In his Preface, Dr. S. gives the following account of his plan:—"Impreffed with the opinion that chemical analyfis, in its prefent improved ftate, is fully adequate to make us acquainted with all thofe foreign contents that give their medical powers to mineral fprings, I have been more minute on this fubject than perhaps might be thought neceffary by the greater number of readers; but as it appears to me probable, that under particular circumftances of dilution and temperature, the actual quantity of foreign matter contained in any water, produces effects by no means proportionate to thofe that might occur under different circumftances, I have thought it fafeft on the whole, to admit a confiderable degree of accuracy on the fubject of chemical analyfis, where the authorities were fuch as to allow of it, in order that on this head at leaft, there fhould be as little error as poffible; and I have not fcrupled occafionally to introduce remarks which will only be intelligible to the chemical reader. The terms of the modern chemical nomenclature have likewife been moftly adhered to, as well as its principles, as they are fuch as are now the moft familiar to thofe who are converfant in this fcience. With regard to the individual mineral fprings which are particularly treated of, they are very few compared to the vaft number which, by acquiring a certain degree of local reputation, have gained a place in the catalogue of medicated waters; and I have only noticed thofe that enjoy a high degree of celebrity, that are interefting from fome peculiarity of compofition, or that ferve to diftinguifh certain claffes of thefe natural bodies, and illuftrate their medical virtues. The reader will fee, that the particulars concerning the chemical analyfis of thefe fprings are extracted from the various detached publications on the individual waters; and fome pains have been taken to reconcile or account for differences of refult, which fometimes happen where more than one authority has been confulted. Thefe publications likewife generally contain remarks on the medical properties of the mineral water, and directions for its ufe, many of of which, being written by excellent practitioners as well as ingenious chemifts, who have long refided on the fpot, afford much matter that is highly valuable to the phyfician who prefcribes thefe remedies, and to the patient who ufes them. This part of the fubject has been felected with care, and is prefented to the reader accompanied with fuch obfervations as appeared to me to be requifite.

"Any general account of the medicinal ufe of mineral fprings would be imperfect, without taking fome notice of the employment of them as a bath, either generally or topically; and as the fingle circumftance of temperature forms the bafis of the moft important diftinctions on this fubject, I have in the latter end of this work, taken into confideration the ufe of the warm and cold bath, in various difeafes.

"The phyfician is frequently called upon to decide for his patient which of the various medicated waters he fhall ufe; whether fea-bathing or a natural chalybeate, be the moft eligible tonic; whether a gouty dyfpeptic habit will be better put in order by the thermal waters of Bath or Buxton, the tonic purgative water of Cheltenham, or by the fulphureous fprings of Harrogate. Without entering minutely into the circumftances of different cafes, I have endeavoured to lay down fuch general indications, and to notice fuch diftinctions, as will affift the practitioner, or even the invalid, who is in fome degree acquainted with thefe fubjects, to make his choice upon folid and judicious grounds; and on this head I have often been able to add to theoretical reafoning, the refult of my own experience for many years.

"Such is the plan of the book which is here fubmitted to the candour of the public; and it appears to me, that the prefent time is that, in which a work of this kind may prove of confiderable fervice, by collecting under one point of view, the leading features of thofe important improvements which chemiftry has been making for many years upon thefe fubjects, particularly with regard to their application to medicine."

The book is divided into feven chapters. In the firft Dr. S. examines the chemical conftitution of fimple water, and fhews that it is a component part of blood, and of all the animal and vegetable fluids and folids.

The fecond chapter is on the foreign contents of mineral waters, and the chemical tefts by which they are diftinguifhed. Thefe impregnations are common air, carbonic acid, hydrogen, fulphurated hydrogen, and azotic gafes; calcareous, magnefian, and aluminous falts; foda and ammonia; alkaline neutral falts; iron and copper; bitumen; filiceous and aluminous earth; and alluvial matters.

The author then examines the waters in common ufe, and recommends the following methods of correcting certain defects in them:

"Both chemical and mechanical means have been ufed with this intention; but it is very feldom that the former can be employed in an adequate degree, without altering the tafte; and thereby rendering the water unfit for drinking, though it may be ufeful for other purpofes. All the earthy falts which would oppofe the folution of foap, may be decompofed by a mild alkali, which will caufe the earth to precipitate, and the addition of fo much alkali, and the converfion of an earthy into a neutral alkaline falt, will not injure the folvent powers of the water.* Simple boiling will likewife foften thofe waters whofe hardnefs confifts in mere carbonated earths, efpecially the calcareous. As this expels the carbonic acid, the earth fubfides. This however will not remove felenite; and as moft hard waters contain both thefe calcareous falts, boiling is only partially ufeful. Another and more extenfively applicable method is that of filtration. The principle of this procefs is, to caufe water that is foul to pafs through the very minute pores of any fubftance not of itfelf capable of imparting any thing to the water; and by this means every thing which is fimply fufpended in the liquor, but fo intimately diffufed as not to be feparable by mere repofe, is detained in the filter, and the water paffes through clear and limpid. This is a procefs which is performed largely within the earth, and hence we find the pureft waters to arife through clear fand or filiceous rock. To imitate this natural procefs, nothing is better than the porous free-ftone of which filtering ftones are ufually made. Sand, clear gravel, pounded glafs, and fimilar fubftances, are equally fitted for this purpofe. Filtration may be performed either by caufing the water to defcend by its own weight through a porous fubftance, or to afcend through it by capillary attraction. The latter method, where it can be adopted, appears preferable. It is to be obferved, however, that common filtration will not render water lefs faline, or feparate any thing that is truly diffolved, but only what is fufpended. I have faid *common* filtration, for it has been fuppofed by fome that fea water, when paffing up through a confiderable ftratum of fand, may be deprived of its falt, as well as the impurities which vifibly foul it. It is certain that in many places remarkably good frefh water is found by digging a few feet in the fand on the fea fhore, at a very fhort diftance from the high-water mark. This is the cafe at Yarmouth, on the Norfolk coaft, and the water procured from thefe wells is purer than any other that is found about the town; but there is no direct evidence that this is fea-water filtered by afcent through the fand, fince it may be well fuppofed to be frefh water rifing from a greater diftance within land, that has undergone the laft degree of purification, by its paffage through the fine clear fand of which the foil is compofed, for a confiderable diftance off the fea fhore.

"Again, there are numberlefs inftances where fprings of frefh water of great purity, unqueftionably arifing from the country higher up,

* The afhes of fern or wormwood, which contain a good deal of carbonat of potafh, are often ufed in various parts of the country for foftening hard water for the purpofe of wafhing.

up, are feen clofe to the fea fhore. Thus at Scarborough the haven is always left dry at low water, and at that time a number of frefh water fprings are detected pouring their contents on the beach which is left bare by the tide. However the poffibility of fweetening falt water by mere filtration be determined, there is no method that we are yet acquainted with of performing this with fufficient eafe and expedition, to be employed for ordinary purpofes. A turbid brine, by paffing through a tub of clean fand, will run through perfectly clear, but quite as falt as before. It appears, however, that an earth fufpended by carbonic acid, as for example, the carbonat of lime, will in a confiderable degree be feparated in the filter, owing to the very divided furface of the water, by which much of this acid will be diffipated; and thus a hard water will fometimes be rendered fofter as well as clearer by filtration."

The fucceeding chapter, which is by far the longeft in the book, gives a particular account of the waters of

| | | | | |
|---|---|---|---|---|
| Malvern | Bath | Tunbridge | Vichy | Aix |
| Holywell | Sedlitz | Spa | Carlfbad | Borfet |
| Briftol | Epfom | Pyrmont | Hartfell | Barege. |
| Matlock | Sea | Cheltenham | Harrogate | |
| Buxton | Seltzer | Scarborough | Moffat | |

Together with a Synoptical Table of the compofition of thofe waters.

The two next chapters treat of the *internal* ufe of water, both as an article of diet and medicine.

The fixth chapter contains defcriptions of, and directions for, the *external* ufe of water, or the different kinds of bathing.

From the concluding chapter we prefent our readers with the following extracts:

"In perufing the moft popular treatifes on particular mineral waters, efpecially on thofe of high and juftly acquired reputation, written by practitioners long refident on the fpot, we fhall find in many of them a great fund of accurate chemical knowledge, and excellent medical obfervations; but we fhall alfo, in the greater number of thefe works, meet with certain modes of treating the fubject, which may fairly be brought under candid criticifm. Some of thefe writers (efpecially thofe who have fhewn themfelves fkilful and zealous chemifts) have, I think, fometimes refined too much on the fcience, and have endeavoured to transfer the fame accuracy of difcrimination which experimental chemiftry affords, to the explanation of minute effects produced on the living body by various fubjects during their ftay in its complicated organs. Others again, have endeavoured to throw a veil of myftery over the whole fubject, and, profeffing to difregard all the information which chemiftry affords, they have ftudioufly avoided any attempt to explain the effects produced by certain mineral waters from a review of their contents, and have ftrongly favoured the idea of their being *fpecifics*, prepared by the hand of nature, againft fome of the moft formidable and obftinate difeafes with which the human race is afflicted.

"It

"It is urged by ſome of the merely practical writers on particular mineral waters, that the mode in which their active contents operate, is equally myſterious with that of ſome of our moſt powerful medicines, ſuch as mercury and opium, and probably likely always to remain ſo; and therefore that accurate chemical examination is little elſe than philoſophical trifling. But it ſhould be remembered, that every medicated water is a compound, at leaſt, of the water itſelf, and of that which gives it its ſenſible properties, and theſe it is the peculiar object of chemiſtry to ſeparate, and to eſtimate their reſpective proportions. Now, no practitioner will deny that it is of conſequence to know the proportional quantity of each ingredient in a compound form of medicine, that he may at leaſt endeavour to aſcribe to each its particular operation, although he does not pretend to explain how that operation is to be brought about.

"The compoſition of the moſt celebrated mineral waters having been known by accurate analyſis, ſeveral ingenious men have endeavoured to confirm their experiments by ſyntheſis; and to produce, by artificial means, a compound, in all reſpects ſimilar to the natural preparation. To this too was added the additional motive of being able to ſupply the want of the natural waters, in places, and at times, when theſe could not be procured. The illuſtrious Bergman, in his excellent Treatiſe on Mineral Waters, has given very good ideas on the method of preparing them artificially; but ſome of the proceſſes which he propoſes, are imperfect, and liable to objections. Where the water to be imitated is only a ſolution of ſome neutral ſalts, ſuch, for inſtance, as the Epſom, Sedlitz, or Sea water, all that it is neceſſary to know, is the proportion in which they are contained in the natural ſpring; but the proceſſes of nature are not always imitated with ſo much eaſe. A greater difficulty lay in the way, which was, that of impregnating water with gaſeous ſubſtances to as complete a ſaturation as is found in ſome of the moſt powerful mineral ſprings. This every chemiſt knows to be a very difficult object to attain, and impracticable with any of the more common apparatus now in uſe for ſuch purpoſes; but, under particular management, it has been attained, and ſome of the ſpecimens of artificially carbonated water that are to be ſeen, appear fully to equal in this reſpect the natural waters of Pyrmont or Seltzer. The ſaline and chalybeate principles may alſo be eaſily added, and the imitation will be complete for all medical purpoſes."

---

*A Practical Treatise on the different Fevers of the West Indies, and their Diagnoſtic Symptoms.* By WILLIAM FOWLE, M. D. 8vo. pp. 93. London, 1800. Symonds.

Dr. F. in common with many of his brethren, had been ſtruck with the diſcordance of medical authors on the ſubject of the fevers of the Weſt Indies; therefore, when he was appointed Phyſician to the forces on that ſtation, he determined to uſe his beſt endeavours to elucidate a ſubject of ſo much national importance.

"Very

"Very early after my arrival in the country, I obferved, that perfons attacked with fever, in almoft any fituation, very generally became yellow. This foon led me to conceive it merely a concomitant fymptom, and by no means fuch as could be fufficiently characteriftic of any one fever, to give it a particular denomination; it alfo led me to difcover the caufe of the variety of fymptoms attributed by different authors to the Yellow Fever, and to account for fuccefsful methods of cure which were often diametrically oppofite to each other. The longer I remained in the country, the more I was convinced of the danger attendant on giving a name to one difeafe from a fymptom common to fo many. An order coming out, that all the medical officers at that time on the Leeward Ifland ftaff fhould deliver in their opinions on the difeafes of the troops in the Weft Indies, together with the moft fuccefsful method of cure, &c. through the Infpector General of the Leeward Ifland Hofpitals, to the Army Medical Board; I conceived I could not better anfwer the purpofe propofed, than by fubmitting to them the obfervations I had made on the different difeafes, laying a confiderable ftrefs on thofe fymptoms that feem to point out more particularly the different fevers one from another; the mode of treatment being fo effentially different, and the fevers of that climate being fo quick in their progrefs, as to make a few hours' delay, for the purpofe of afcertaining their tendency to remiffion, productive of the moft imminent danger. In doing this, I found more matter than I expected prefs on me; and though I began with an intention of comprifing all I had to fay in a few fheets, I found at the conclufion that I had written a little volume. On my return to England, having frequent occafion to converfe with fome medical friends on the fubject of thofe difeafes which I had been moft in the habit of obferving, and finding it difficult to explain my ideas as perfectly as I wifhed in converfation, I put a copy of the work into their hands. The obfervations there contained were conceived by them to lead to fome important points of practice. I was advifed and defired to make fuch additions as might render them more inftrumental to the fervice of thofe who are likely to be engaged in the fame line, and to offer them to the perufal of the public.

"I have now complied with thofe defires; and at the end of this work, I have alfo added a few prefcriptions for the ufe of young men who may be fent to that country, immediately on entering the army. I am well convinced, that fuch formulæ, having nothing very particular in them, might, for the generality of readers, have been well omitted: but, from experience, I feel myfelf authorifed to fay, that many young men, who may undergo very ftrict examinations on the fubjects of anatomy, furgery, and the fymptoms and general cure of difeafes, have ftill much to learn as to the compofitions of medicines and their proper dofes; and I here beg leave earneftly to requeft a more proper degree of attention to that fubject."

Dr.

Dr. F. divides the fevers of the Weft Indies into intermittents, remittents, ardent fever, and the malignant or jail fever. On the diagnoftic fymptoms of thefe varieties of fevers, he fays,

"It may affift the reader if we bring together what has been fcattered in the former chapters concerning the different fituations, feafons of the year, and predifpofitions, which conduce to the production of the various fevers.

"We may obferve that the ardent fever requires a certain degree of firmnefs of fibre: it reigns moft commonly at that period of the year when the fky is clear; when the atmofphere feems little, if at all, loaded with vapour, and when the heat is great. The objects of its attack are the ftout and athletic, young or middle aged men, or women who in their conftitutions and habits nearly refemble them, and thofe who have lately arrived either from Europe or North America, or from the more mountainous fituations in the Weft Indies themfelves. It is prevalent in the dry fandy bays, and is often induced by perfons who have been much heated being fuddenly expofed to cold.

"The remitting fever, on the contrary, feems to require a previoufly debilitated body: it is moft frequent at thofe periods of the year, when, the ground having been fuperfaturated by the rains, the whole atmofphere becomes loaded with noxious vapour. Perfons who have lived for fome time in the Weft Indies are by no means freed from its power, and women and children fuffer feverely from it, as do alfo thofe who have been debilitated by previous illnefs or long continued fatigue. It delights in the low fwampy marfhes, in the neighbourhood of lagoons, in uncultivated fituations, where the currents of air are impeded, and on the fmaller hills which are fubject alternately to the fwampy vapours, and the cold ftorms.

"The gaol fever is feldom to be met with except on board of fhips or in crouded towns, or in individuals who have been expofed to the contagion of fuch places.

"This recapitulation may lead us to a very important point of practice; it may teach us to be very minute in our enquiries into the former manner of living of our patient, and alfo into his previous refidence and other circumftances: for it muft appear to be the height of imprudence to bleed a man profufely, and to have recourfe to other violent evacuations to a very great extent, though he fhould be young in appearance, robuft, and attacked in a dry hot fituation, when we might find, by a more minute enquiry, that this man has, for weeks previous to his illnefs, been undergoing immenfe fatigue, in fituations too the moft expofed to fwampy vapours, and perhaps with but fpare diet and little fleep. Nor is this fuppofing a cafe merely for the fake of illuftration; the thing itfelf is continually occurring. Small detachments from regiments are frequently fent not only from one part of an ifland to another, but alfo to different iflands; foldiers thus detached are very apt to be feized with fevers, not only from change of air, which I have before afferted to be a very frequent exciting caufe, but but alſo from having greater opportunities of obtaining ſpirits. The danger of following this evacuating plan in a man who has been expoſed to the influence of contagion muſt be ſtill more evident.

"On the contrary, where the patient happens to be attacked ſoon after he comes into the neighbourhood of ſwampy ſoils, not having before been expoſed to any debilitating cauſes, we ſurely may be more free in our evacuations, than in a more weakened perſon, or one who had been a longer reſident.

"We may obſerve that the mode of attack differs conſiderably in the three fevers. While the gaol fever is commonly preceded, for ſome hours, often for a day or two, by languors and tranſient alternations of chills and fluſhes, and the remitting has a *rigor*, generally ſtrong but always ſufficiently evident, the ardent fever commonly makes its attack of a ſudden, and with little or no *rigor*, and the patient, from being in apparent ſound health, is hurried inſtantly into the midſt of diſeaſe. The proſtration of ſtrength alſo, though very great both in the remitting and typhus, yet bears no proportion to that in the ardent: the pain in the head, back, and limbs is conſiderable in all, but in the ardent there is moſt commonly a violent pain in the middle of the thighs. The pain alſo of the head is different in the various fevers: in the ardent, it is principally fixed over the orbit of each eye; while there is only a dull heavy pain over the reſt of the head: in the remitting, the pain is violent and continued over the whole head; while in the gaol fever it ſeems rather to be a ſucceſſion of pulſations, giving an idea to the patient that his head is forcibly opening and ſhutting. There is little vomitting, except juſt in the early attack of gaol fever, but in both the others, it is a troubleſome and dangerous ſymptom: in the remitting, there is generally a quantity of bile thrown up, frequently of a green colour, but this ſeldom or never happens in the ardent. So conſtantly indeed is this the caſe, that where bile is vomitted up, and there has been ſenſible *rigor*, I ſhould have ſcarcely any doubt in pronouncing the fever not to be of the ardent type: and there is alſo in the latter a ſenſation of burning at the pit of the ſtomach.

"The countenance in the gaol fever is commonly, although ſlightly ſuffuſed, yet of a dirtyiſh hue, and by no means tumid, and there is commonly violent pulſation of the carotid arteries. In the remitting, after the hot fit is formed, the ſuffuſion is great, but the tumour by no means ſo evident, particularly about the fauces and neck, as in the ardent. The mode of ſpeaking alſo is very different: in this, it is confuſed and thick; in the remitting, it varies only from health in a quickneſs, owing to the anxiety of the patient, not to any particular debility of the organs of ſpeech; and in the gaol fever it is generally a quickneſs of ſpeech for a word or two, rather as if from impatience at being diſturbed, or elſe it is plaintive and querulous. Neither in the remitting nor typhus has the patient any of that appearance as of intoxication, which is frequently to be obſerved in the ardent fever.

"The tongue in typhus is generally very tremulous when put out: it is from the beginning furred, and foon becomes dry and chapped; and, towards the latter ftages of the difeafe, the tongue and lips are covered with black and loofe faburra, floating like cobwebs, or they have a number of apthæ over their furface. In the remitting, the tongue is furred, but in no degree, and, as the difeafe advances, this fur becomes brown, but there is in general fome moifture on it to the laft. In the ardent fever the tongue has no fur upon it; on the contrary, we may call it morbidly clean: it is rather moift, and of a bright red colour: as the difeafe advances, it fometimes becomes dry, at others not fo, but always continues clean. The thirft is not fo great as we might be led to expect in this fever, from the vehemence of the fymptoms, neither is it in any proportion to what is felt in the others. In this fever alfo, while the proftration of ftrength is very great, the vomiting, burning heat of the ftomach, and reftleffnefs are violently urgent, and the heat of the fkin intenfe. The pulfe varies very inconfiderably, either in ftrength or quicknefs from its natural ftate, but in the other fevers, it is amongft the firft fymptoms to fhew a derangement of the fyftem.

"The delirium is very different alfo: in the ardent fever, it is of the moft fierce kind. The patient generally imagines himfelf in the prefence of his moft bitter enemies, who are either attacking him, or whom he is endeavouring to attack; the eye-balls are ftrained, the whole countenance puts on the moft terrific afpect, and it often requires two or three perfons to hold him in his bed. The delirium in the remitting is feldom fo fierce; it is generally mild, the imagination of the patient bufying itfelf with his former occupations and purfuits; while that of typhus feems fcarcely to amount to more than a want of power of attention in the fick perfon to any thing about him; for during the time he is uttering the moft incoherent nonfenfe, if he be roufed, he gives a rational anfwer, but immediately relapfes into his incoherent fit.

"Thefe are the fymptoms which are moft diffimilar in the different fevers. I have purpofely omitted the yellownefs of the fkin, the black vomit, and the hæmorrhages from different parts of the body, as I have feen them occur in every fpecies of fever in the tropical climates."

---

*A Treatife on Opthalmy, and thofe Difeafes which are induced by Inflammations of the Eyes, with new Methods of Cure. Part the Firft. By* EDWARD MOORE NOBLE, Surgeon, 8vo. pp. 144. London, 1800, Robinfons.

THE Author's reafons for appearing before the public on this fubject, are thus ftated by him:—

"The art of curing difeafes has, of late years, made rapid ftrides towards arriving at that *acmé* of perfection, beyond which it is not in the power of man to advance it. The light which has been let in upon us, by the doctrines of that great genius, Dr. John Brown,

Brown, and fo ably feconded by a Darwin, and a Beddoes, has laid the foundation of a new æra in the annals of medicine, and has opened new views to the practitioner, in the theory and treatment of difeafes.

"Since that epoch no regular treatife, on the inflammation of the eye, has appeared, in which the mode of treatment has been founded on fyftematic principles; and when we confider the importance and delicacy of that organ, we fhall be furprifed to find, how little has been done, towards arriving at a rational plan of cure.

"The regular routine of bleeding, blifters and cathartics, with a variety of external applications, are generally indifcriminately applied, with little attention to the nature, or the period of the difeafe; and there are fome practitioners who act with ftill greater inconfiftency, and maintain, that it is ufelefs to infpect the eye; that it caufes an unneceffary degree of irritation; and that we ought to truft entirely to internal medicines.

"It is my intention to endeavour to point out the propriety of a certain mode of treatment, and to difcriminate the proper time for the ufe of different applications.

"On the firft attack of an Opthalmy, the general circulation of the mafs of fluids is not evidently increafed, though it may be fo in a fhort time, from the pain and irritation of the eye; I therefore confider the difeafe as a topical affection, and that we ought chiefly to rely on local applications for a cure.

"In fpeaking of the caufes of Opthalmy, I have divided them into an increafe of ftimulus, or an increafe of that fomething to which I have given the name of the irritable principle; but whether it ought to be confidered as a quality of vitality, or as fupplied by the brain, or from the atmofphere, I have not ventured to rifk an opinion.

"It probably may be thought, for a Treatife on the Opthalmy, I have entered into too long a digreffion upon the effects of *ftimuli* applied to the body, and have been too prolix upon the laws relative to the accumulation or exhauftion of this irritable principle. Much more might undoubtedly have been faid, which would have better elucidated the doctrine, but I wifhed not to take up too much of my reader's time upon the difcuffion, and I knew not how, in faying lefs, to properly explain my ideas on thofe fubjects.

"It may, perhaps, not be improper in this place, to take a general view of what is intended to be given in the Second Part, which will conclude this Treatife.

"In the firft place I fhall enter upon the cure of the inflammation of the eye.

"In the laws of the animal economy, there is fcarce any fact more clear than a *ftimulus* ftronger than ufual being applied to the moving fibre, makes it lefs eafily excited into action, and that on the fudden fubftraction of this increafed *ftimulus*, the motions of the part will be diminifhed.

"Upon this law will depend my method of cure of the Opthalmy. The treatment of the difeafe admits of a variety of modifications; difications; but my principal object will be the application of a *stimulus*, in a peculiar manner, as great as the eye can bear, without being thrown into convulsive motions, and when this *stimulus* loses its effect of causing pain, to suddenly remove it, and diminish all *stimuli*, or irritating causes, as much as possible.

"By these means a diminished action of the vessels will be induced, the pain will be moderated, and an alleviation of the symptoms will take place.

"I hope to be able to send the Second Part, which will conclude this Treatise, to the press the beginning of next year."

The above extract will enable our readers to form a judgment of the Author's stile and principles of practice.

---

### *Mr. Davy's Chemical and Philosophical Researches.*

[ Continued from pp. 265—269, of our last. ]

The following experiments are a proof of Mr. D's intrepidity, and of the deleterious effects produced by the respiration of hydrocarbonate.

"In the first experiment I breathed for near a minute, three quarts of hydrocarbonate mingled with nearly two quarts of atmospheric air. It produced a slight giddiness and pain in the head, and a momentary loss of voluntary power; my pulse was rendered much quicker and feebler. These effects, however, went off in five minutes, and I had no return of giddiness.

"Emboldened by this trial, in which the feelings were not unlike those I experienced in the first experiments on nitrous oxide, I resolved to breathe pure hydrocarbonate.

"For this purpose, I introduced into a silk bag, four quarts of gas nearly pure, which was carefully produced from the decomposition of water by charcoal an hour before, and which had a very strong and disagreeable smell.

"My friend, Mr. James Tobin, jun. being present, after a forced exhaustion of my lungs, the nose being accurately closed, I made three inspirations and expirations of the hydrocarbonate. The first inspiration produced a sort of numbness and loss of feeling in the chest and about the pectoral muscles. After the second inspiration, I lost all power of perceiving external things, and had no distinct sensation, except a terrible oppression on the chest. During the third expiration, this feeling disappeared, I seemed sinking into annihilation, and had just power enough to drop the mouth-piece from my unclosed lips. A short interval must have passed, during which I respired common air, before the objects about me were distinguishable. On recollecting myself, I faintly articulated, '*I do not think I shall die.*' Putting my finger on the wrist, I found my pulse thread-like, and beating with excessive quickness.

"In less than a minute I was able to walk, and the painful oppression on the chest directed me to the open air.

"After making a few steps, which carried me to the garden, my my head became giddy, my knees trembled, and I had juſt ſufficient voluntary power to throw myſelf on the graſs. Here the painful feeling of the cheſt increaſed with ſuch violence, as to threaten ſuffocation. At this moment, I aſked for ſome nitrous oxyde. Mr. Dwyer brought me a mixture of oxygene and nitrous oxyde. I breathed this for a minute, and believed myſelf relieved. In five minutes, the painful feelings began gradually to diminiſh; in an hour they had nearly diſappeared, and I felt only exceſſive weakneſs and a ſlight ſwimming of the head. My voice was very feeble and indiſtinct. This was at two o'clock in the afternoon.

"I afterwards walked ſlowly, for about half an hour, with Mr. Tobin, jun. and on my return was ſo much ſtronger and better, as to believe that the effects of the gas had diſappeared; though my pulſe was 120, and very feeble. I continued without pain for near three quarters of an hour, when the giddineſs returned with ſuch violence as to oblige me to lie on the bed; it was accompanied with nauſea, loſs of memory, and deficient ſenſation. In about an hour and a half the giddineſs went off, and was ſucceeded by an excruciating pain in the forehead and between the eyes, with tranſient pains in the cheſt and extremities.

"Towards night theſe affections gradually diminiſhed. Between eight and ten, I took, by the advice of Dr. Beddoes, two or three doſes of diluted nitric acid. At ten, no diſagreeable feeling except weakneſs remained. I ſlept ſound, and awoke in the morning very feeble and very hungry. No recurrence of the ſymptoms took place, and I had nearly recovered my ſtrength by the evening.

"I have been minute in the account of this experiment, becauſe it proves, that hydrocarbonate acts as a ſedative; i. e. that it produces diminution of vital action, and debility, without previouſly exciting. There is every reaſon to believe, that if I had taken four or five inſpirations inſtead of three, they would have deſtroyed life immediately, without producing any painful ſenſation.—Perhaps moſt of the uneaſy feelings after the experiment, were connected with the return of the healthy condition of organs.

"About a week after this experiment, I attempted to reſpire carbonic acid, not being at the time acquainted with the experiments of Roſier.

"I introduced into a ſilk bag four quarts of well waſhed carbonic acid produced from carbonate of ammonia by heat; and after a complete voluntary exhauſtion of my lungs, attempted to inſpire it. It taſted ſtrongly acid in the mouth and fauces, and produced a ſenſe of burning at the top of the uvula. In vain I made powerful voluntary efforts to draw it into the wind-pipe; at the moment that the epiglottis was raiſed a little, a painful ſtimulation was induced, ſo as to cloſe it ſpaſmodically on the glottis; and thus, in repeated trials, I was prevented from taking a ſingle particle of carbonic acid into my lungs.

"I tried

"I tried to breathe a mixture of two quarts of common air and three of carbonic acid, without ſucceſs; it ſtimulated the epiglottis nearly in the ſame manner as pure carbonic acid, and was perfectly non-reſpirable.

"I found that a mixture of three quarts of carbonic acid with ſeven of common air was reſpirable; I breathed it for near a minute. At the time, it produced a ſlight degree of giddineſs, and an inclination to ſleep. Theſe effects, however, very rapidly diſappeared after I had ceaſed to breathe, and no other affections followed.

"During the courſe of experiments on nitrous oxyde, I ſeveral times breathed oxygene procured from mangaueſe by heat, from three to five minutes.

"In reſpiring eight or ten quarts; for the firſt two or three minutes I could perceive no effects. Towards the end, even when I breathed very ſlowly, my reſpiration became oppreſſed, and I felt a ſenſation analogous to that produced by the want of freſh air; though but little of the oxygene had been conſumed.

"In one experiment, when I breathed from and into a bag containing twenty quarts of oxygene for near ſix minutes, Doctor Kinglake felt my pulſe, and found it not altered in velocity, but rather harder than before. I perceived no effects but thoſe of oppreſſion on the cheſt.

"Having obſerved in my experiments upon venous blood, that nitrous gas rendered that fluid of a purple tinge, very like the colour generated in it by nitrous oxyde; and finding no painful effects produced by the application of nitrous gas to the bare muſcular fibre, I began to imagine that this gas might be breathed with impunity, provided it were poſſible in any way to free the lungs of common air before inſpiration, ſo as to prevent the formation of nitrous acid.

"On this ſuppoſition, during a fit of enthuſiaſm produced by the reſpiration of nitrous oxyde, I reſolved to endeavour to breathe nitrous gas.

"One hundred and fourteen cubic inches of nitrous gas were introduced into the large mercurial airholder; two ſmall ſilk bags of the capacity of ſeven quarts were filled with nitrous oxyde.

"After a forced exhauſtion of my lungs, my noſe being accurately cloſed, I made three inſpirations and expirations of nitrous oxyde in one of the bags, to free my lungs as much as poſſible from atmoſpheric oxygene; then, after a full expiration of the nitrous oxyde, I transferred my mouth from the mouth-piece of the bag to that of the airholder, and turning the ſtop-cock, attempted to inſpire the nitrous gas.—In paſſing through my mouth and fauces, it taſted aſtringent and highly diſagreeable; it occaſioned a ſenſe of burning in the throat, and produced a ſpaſm of the epiglottis, ſo painful as to oblige me to deſiſt inſtantly from attempts to inſpire it. After moving my lips from the mouth-piece, when I opened them to inſpire common air, aëriform nitrous acid was inſtantly formed in my mouth, which burnt the tongue tongue and palate, injured the teeth, and produced an inflammation of the mucous membrane, which lasted for some hours.

"As after the respiration of nitrous oxyde in the experiments in the last Research, a small portion of the residual atmospheric air remained in the lungs, mingled with the gas, after forced expiration; it is most probable that a minute portion of nitrous acid was formed in this experiment, when the nitrous gas was taken into the mouth and fauces, which might produce its stimulating properties. If so, perhaps I owe my life to the circumstance; for supposing I had taken an inspiration of nitrous gas, and even that it had produced no positive effects, it is highly improbable, that by breathing nitrous oxyde, I should have freed my lungs from it, so as to have prevented the formation of nitrous acid when I again inspired common air. I never design again to attempt so rash an experiment.

"In the beginning of September I often respired nitrous oxyde mingled with different proportions of common air or oxygene. The effects produced by the diluted gas were much less violent than those produced by pure nitrous oxyde. They were generally pleasant: the thrilling was not often perceived, but a sense of exhiliration was almost constant.

"Between September and the end of October, I made but few experiments on respiration, almost the whole of my time being devoted to chemical experiments on the production and analysis of nitrous oxyde.

"At this period my health being somewhat injured by the constant labour of experimenting, and the perpetual inhalation of the acid vapours of the laboratory, I went into Cornwall; where new associations of ideas and feelings, common exercise, a pure atmosphere, luxurious diet, and moderate indulgence in wine, in a month restored me to health and vigor.

"Nov. 27th. Immediately after my return, being fatigued by a long journey, I respired nine quarts of nitrous oxyde, having been precisely thirty-three days without breathing any. The feelings were different from those I had experienced in former experiments. After the first six or seven inspirations, I gradually began to lose the perception of external things, and a vivid and intense recollection of some former experiment passed through my mind, so that I called out, "*what an amazing concatenation of* "*ideas!*" I had no pleasurable feeling whatever, I used no muscular motion, nor did I feel any disposition to it; after a minute, when I made the note of the experiment, all the uncommon sensations had vanished; they were succeeded by a slight soreness in one of the arms and in the leg: in three minutes these affections likewise disappeared.

"From this experiment I was inclined to suppose that my newly acquired health had diminished my susceptibility to the effects of the gas. About ten days after, however, I had an opportunity of proving the fallacy of this supposition.

"Immediately after a journey of 126 miles, in which I had no sleep fleep the preceding night, being much exhaufted, I refpired feven quarts of gas for near three minutes. It produced the ufual pleafurable effects, and flight mufcular motion. I continued exhilarated for fome minutes afterwards: but in half an hour found myfelf neither more or lefs exhaufted than before the experiment. I had a great propenfity to fleep.

"I repeated the experiment four or five times in the following week, with fimilar effects. My fufceptibility was certainly not diminifhed; I even thought that I was more affected than formerly by equal dofes.

"Though, except in one inftance, when indeed the gas was impure, I had experienced no decifive exhauftion after the excitement from nitrous oxyde, yet ftill I was far from being fatisfied that it was unanalogous to ftimulants in general.—No experiment had been made in which the excitement from nitrous oxyde had been kept up for fo great a length of time, and carried to fo great an extent as that in which it is uniformly fucceeded by exceffive debility under the agency of other powers.

"It occurred to me, that fuppofing nitrous oxyde to be a ftimulant of the common clafs, it would follow that the debility produced in confequence of exceffive ftimulation by a known agent, ought to be *increafed* after excitement from nitrous oxyde; in the fame manner as the debility from intoxication by two bottles of wine is increafed by a third.

"To afcertain whether this was the cafe, I made on December 23d, at four P. M. the following experiment: I drank a bottle of wine, in large draughts, in lefs than eight minutes. Whilft I was drinking, I perceived a fenfe of fulnefs in the head, and throbbing of the arteries, not unanalogous to that produced in the firft ftage of nitrous oxyde excitement. After I had finifhed the bottle, this fulnefs increafed, the objects around me became dazzling, the power of diftinct articulation was loft, and I was unable to walk fteadily. At this moment the fenfations were rather pleafurable than otherwife, the fenfe of fulnefs in the head foon however increafed fo as to become painful, and in lefs than an hour I funk into a ftate of infenfibility. In this fituation I muft have remained for two hours or two hours and a half."

[ To be continued. ]

---

*De la Pefte, ou les Epoques memorables de ce Fleau, i. e.* On the Plague, or the memorable Epochs of this Evil, and the Means of preventing it. By J. P. Sapon, &c. Paris, for Lavilette and Co. 2 vol. 8vo. pp. 300.

The meafures which are taken in the different ports of the Mediterranean, to prevent the infection of the plague, may be fufficient in time of peace; but in time of war, where the general police can be eafily difturbed, and the laws on which the welfare of nations tions rely, fometimes neglected by circumftances, it would undoubtedly be very imprudent to confider ourfelves equally fecure. In the general agitation of things in which we now live, this fcourge of mankind may fteal into Europe in more than one way. The different means to import fmuggled wares may, particularly at this time, open a way to this evil, to pafs the barriers which are oppofed to it; and there are likewife inftances of its having efcaped from military hofpitals under the apparent fymptoms of a common epidemic difeafe, when it afterwards affumed the character of a true plague.

Amidft fo many critical and dangerous circumftances, *Cit. Sapon* has endeavoured to ferve the community, by collecting every thing that experience and obfervation have taught the beft informed phyficians, or the perfons that fuperintended the execution of thofe laws for preventing the plague. After having, in the introduction, made fome reflections on the origin and caufes of the plague, he proceeds to give, in the *firft* volume, the hiftory of this evil, confidered in its moft remarkable epochs; he fpeaks of the plague of *Athens* in the year 335, before the Chriftian era; of that of *Conftantinople* in the year of our Lord 542; of that in 1347, otherwife called the *black plague*; of that of *Milan*, 1629 and 30; of that of *Lyons*, 1628 and 29; of that of *Montpelier*, 1629; of that of *Digue*, 1629; of that of *Marfeille*, 1720; and of that of *Toulon* and *Aix* in the fame year.

By prefenting thus to the reader the ravages of the plague, its accidents, fymptoms, and character, by conducting him to the difmal fcenes of a defolate city, and of a perfon afflicted with the plague, and ftruggling againft pains and inevitable death, the author attempts to awaken the attention of Governments and individuals, to caution themfelves with every poffible care againft the moft infidious and dangerous enemy of mankind; and he concludes this volume with giving an account of the fteps and meafures that were taken in thofe dangerous times.—We do not fuppofe that candid readers will expoftulate with the author for only having given the alarm, by reprefenting this picture of mifery and defolation, occafioned by the plague; and had he publifhed his work without adding the adminiftrative and preventive part, he might have deferved this reproach. Such a falfe delicacy, however, may fometimes do much harm, by hiding from us the danger, and of courfe preventing us from taking proper meafures.—In the *fecond volume*, he treats of the laws which ought to be eftablifhed in all fea ports, where there is the greateft danger of the plague being imported. The whole terminates with an hiftorical chronology of the plagues that ravaged Europe before and after the Chriftian æra.

---

*Archive für die Medicinifche Landerkunde, i. e.* Archives for the Medical Knowledge of Countries, Vol. I. *No.* 1. Koburg & Leipzic, for J. C. SINNER, 8vo. 1800.

THE Author profeffes this firft number of a new periodical publication, devoted to the medical knowledge of countries, to be nothing but a trial of the kind, to fee whether the plan conceived by the author author of collecting medico-topographical notices of single countries and places, which are here and there scattered, will meet with the approbation of the public. He gives in this number fragments of medical topographies extracted from different known Journals, that of *Hufeland*, &c. The places he acquaints us with, are different towns of Germany, as *Clausthal*, on the Harz forest, *Weymar*, &c. to which is added a description of the hospital at *Bamberg*, one of the best in Germany.—The interest of such an undertaking, as well for the physician as the geographer, appears evident; and it is to be wished, that the Editor may be sufficiently supported, to enable him to enlarge his plan, and to conduct and execute it properly.

---

*Erfahrungen über den Runkel ruben Zucker, i. e.* Experiments on the sugar of the Beet Root, with some ideas and proposals on its fabrication upon a large scale, and on the culture of the beet root.—By W. A. Lampsadius, Professor of chemistry at Freyberg. 1800. (8vo. pp. 84.) Freyberg, for Cratz, price 12 groshen, or nearly 2s.

This pamphlet is one of the best that has appeared on the subject. The experiments are made with beet roots, cultivated both in the common way and after the method of Achard; but the proportion of sugar that is obtained varies according to the soil wherein they grow. The land required for their culture, to make them productive of sugar, must be loose, easily drying, and well manured, and at the same time be sufficiently exposed to the action of the sun. By a chemical analysis, he found them to contain, of water, from 92 to 94 per cent. sugar 2—3½ per cent. mucous matter, 2—3, of albuminous and farinaceous matter, but a small quantity, besides some odorate; and in the yellow variety some colouring principle and a bitter tasting *fixed* matter, &c. He then relates his procedures for obtaining sugar. The juice, after the roots have been ground, is expressed in a strong bag of horse-hair, and boiled for 10 to 15 minutes, with the addition of charcoal powder and as much lime, till the blue vegetable colour is no more changed by the juice, while it is continually stirred and the scum taken off. After this, it is filtered warm through flannel, and quickly cooled by pouring it into flat vessels, and afterwards clarified in a proper vessel with milk, that has been before fleeted. It is then, after 24—36 hours, again poured into a flat copper vessel, which is well tinned within, and exposed to evaporate at 50° of *Reaumur*, till one-third of the juice is gone, and it has obtained the consistency of a thin syrup. Let it now stand for 48 hours till it be cooled, and begin the evaporation again at 40° in flat dishes, till a cuticle appears on the surface of it. In this way the crystals appear in the greatest quantity, and the mucous matter separates a great deal better. The crystals are at last expressed, the syrup distilled for making brandy, and the waste parts used for feeding cattle. The juice is made stronger by frost, and requires afterwards not so much fire. After having mentioned something about the establishment ment of fugar works, he concludes with propofing different mixtures of earths in which the beet roots may beft profper, and with giving an account of the expences and profit of 100 quintals of the beet root, when treated for obtaining fugar, fyrup, and brandy from them.

---

*Beytrag zur Beurtheilung,* i. e. Contribution to a Criticifm of the Brunonian Syftem of Medicine, and the late Refearches upon it; by Dr. CAPPEL, fecond edition, 1800, pp. 424, 8vo. Gottingen, Dieterich.

THE firft edition of this well written work, containing 142 pages, appeared in 1797; the prefent is therefore confiderably augmented, and in many refpects altered. The author profeffes in this, a greater tendency to Brunonian principles than in the former, and retracts many objections he thought proper at that time to maintain. In the introduction, he ftates the rules by which a fyftem of medicine ought to be judged. Medicine, as an empirical fcience, can never aim at a ftrict fcientific perfection; and a fyftem of it deferves fufficient credit, if it unites under certain principles all facts, with which experience has fupplied us. But as experience and obfervation, the true fources of a practical fyftem, are continually increafing, it is likewife probable, that this may produce changes in the fyftem. Imperfection is therefore a charge by no means fufficient to fubvert a fyftem, which in general anfwers the following requifites: 1. That it propofes general principles as fundamental, abftracted from experience. 2. That thefe general principles are applicable to all poffibles cafes. 3. That the applications deduced from thofe general principles are juft and confiftent. 4. That they are ufeful in practice. After having previoufly given a fketch of Brown's principles, the author proceeds to the criticifm in the following chapters: Chap. 1. *On Brown's definition of life.* Chap. 2. *On Brown's excitability.* Chap. 3. *On Brown's principles with refpect to the ftimuli, as acting upon living bodies.* Chap. 4. *On the laws ftated by Brown for excitability and excitement.* Chap. 5. *On Brown's definition of the different ftates of life.* Chap. 6. *On Brown's diagnoftic.* Chap. 7. *On Brown's materia medica.* Chap. 8. *On Brown's therapeutic, and the examination of the Brunonian fyftem in general,* which the author concludes as follows: "After all, we can affert, that the principles of the Brunonian Syftem are almoft fatisfactory, and the fyftem itfelf does, with few exceptions, anfwer the requifites of a fyftem of medicine." How far this may be allowed, we leave our readers to decide.

---

*Ueber die Pulfader Gefchwulfte,* i. e. On Aneurifms and their Surgical Treatment; by Dr. AUG. FR. AYRER, 1800, 8vo. with a plate. Gottingen, Dieterich.

THIS publication comprifes every thing that has been written on Aneurifms, their different fpecies, caufes, and fymptoms, which

 the the author has faithfully collected, and to which he has added, the accurate anatomical description of those vessels in which that disease frequently occurs. The author afterwards proceeds to give a critical account of the different methods of treatment and operations used in that disease. Now and then he has interspersed his own observations, made in the hospitals of Italy and France; and proposed an improvement of two instruments for the compression and ligature of the arteries, by which only the vessel is pressed, and not the whole limb. On the whole, it is a very useful compilation, as it contains a complete history of an interesting surgical disease, by which much time is saved to any one, who means to read every thing that has been written on it in different countries.

---

*Plantae rariores Hungariae indigenae Descriptionibus et Iconibus illustratae a comite Francisco Waldstein, Caes. Reg. Camerario et ordinis Melitensis equite et Paulo Kitaibel, M. D. Chem. et Bot. Prof. Pesthin,* Decas I. II. III. 1800, gr. folio, pp. 28, and 50 coloured plates, price 15 florins each Decas, or about 1*l.* 5*s.* Vienna, Schauenburg.

It is with pleasure we announce this publication, which is certainly very interesting to a botanist. Of all parts of Europe, the extensive kingdom of Hungary was hitherto but very little, if not at all, botanically searched, and we only know a few plants of that country from the celebrated Mr. Jacquin. Its extent, however, and variety of soil, produces an astonishing variety of vegetables, and many plants. Siberian, Grecian, Piedmontese, Austrian, and even some African, grow here spontaneously, together with some new ones, and the more common plants of Europe. The authors have some years made, and are still making, excursions towards all directions of this kingdom, for the sake of collecting its vegetable productions; of which they intend to figure and describe the rarer and new species. The work is to be published by decades, of which ten will make a volume: the plates are neatly engraved and elegantly coloured, almost in the stile of Jacquin's works, which, however, they surpass in many respects: the descriptions seem to be accurate. The present three decades contain the following rare and new plants, viz. *Lactuca sagittata, Achillea ligulata, Lepidium crassifolium, Scabiosa longifolia, banatica, corniculata; Symphytum cordatum, Alyssum murale, Silene longiflora, Vinca herbacea, Carduus radiatus, Phyteuma canescens, Nymphaea lotus, Cineraria sibirica, Allium atropurpureum, Saxifraga hieracifolia, Crepis rigida, Peucedanum arenarium, Aster canus, &c.*

